# How Accurate Is the Prediction of Maximal Oxygen Uptake with Treadmill Testing?

**DOI:** 10.1371/journal.pone.0166608

**Published:** 2016-11-22

**Authors:** John R. Wicks, Neil B. Oldridge

**Affiliations:** 1 Department of Rehabilitation, Robina Hospital, Robina, Queensland, Australia; 2 College of Health Sciences, University of Wisconsin-Milwaukee, Milwaukee, Wisconsin, United States of America; University of Alabama at Birmingham, UNITED STATES

## Abstract

**Background:**

Cardiorespiratory fitness measured by treadmill testing has prognostic significance in determining mortality with cardiovascular and other chronic disease states. The accuracy of a recently developed method for estimating maximal oxygen uptake (VO_2peak_), the heart rate index (HRI), is dependent only on heart rate (HR) and was tested against oxygen uptake (VO_2_), either measured or predicted from conventional treadmill parameters (speed, incline, protocol time).

**Methods:**

The HRI equation, METs = 6 x HRI– 5, where HRI = maximal HR/resting HR, provides a surrogate measure of VO_2peak_. Forty large scale treadmill studies were identified through a systematic search using MEDLINE, Google Scholar and Web of Science in which VO_2peak_ was either measured (TM-VO_2meas_; n = 20) or predicted (TM-VO_2pred_; n = 20) based on treadmill parameters. All studies were required to have reported group mean data of both resting and maximal HRs for determination of HR index-derived oxygen uptake (HRI-VO_2_).

**Results:**

The 20 studies with measured VO_2_ (TM-VO_2meas_), involved 11,477 participants (median 337) with a total of 105,044 participants (median 3,736) in the 20 studies with predicted VO_2_ (TM-VO_2pred_). A difference of only 0.4% was seen between mean (±SD) VO_2peak_ for TM- VO_2meas_ and HRI-VO_2_ (6.51±2.25 METs and 6.54±2.28, respectively; p = 0.84). In contrast, there was a highly significant 21.1% difference between mean (±SD) TM-VO_2pred_ and HRI-VO_2_ (8.12±1.85 METs and 6.71±1.92, respectively; p<0.001).

**Conclusion:**

Although mean TM-VO_2meas_ and HRI-VO_2_ were almost identical, mean TM-VO_2pred_ was more than 20% greater than mean HRI-VO_2_.

## Introduction

When assessed as oxygen consumption (VO_2_), cardiorespiratory fitness (CRF) may be measured either using a treadmill with conventional gas analysis equipment (TM-VO_2meas_) or predicted from equations based on treadmill speed, incline or treadmill time (TM-VO_2pred_)[1]. The prognostic importance of CRF has been extensively investigated in recent meta-analyses confirming the strong inverse relationships between CRF and all-cause mortality in healthy individuals [2] and in patients with either coronary artery disease (CAD) or congestive heart failure (CHF) [3–6]. The prospective studies included in these reviews involve large numbers of subjects and have shown that a 1 MET (equal to 3.5 mL O_2_ ·kg^-1^·min^-1^) increment increase in CRF is associated with an approximate 10–20% reduction in all cause and cardiovascular mortality [2,7] with a similar effect being observed with CHF [6,8].

Logistics of large studies necessitate prediction of peak VO_2_ (VO_2peak_) as measurement of VO_2_ is costly and time consuming. Equations have been determined for the various treadmill protocols based on the variables of treadmill speed, incline or the test time for a particular protocol, a common reference being ACSM publications [1]. However, many factors may contribute to the error of TM-VO_2pred_. They include 1) treadmill handrail support [9–13], 2) failure to use population specific equations [14–18], 3) inappropriate testing protocol [19–21], 4) delayed oxygen kinetics [22–24], 5) reproducibility of cardiopulmonary parameters [25,26], 6) altered mechanical efficiency with treadmill walking [27] and 7) lack of treadmill calibration [28].

**Fig 1:**
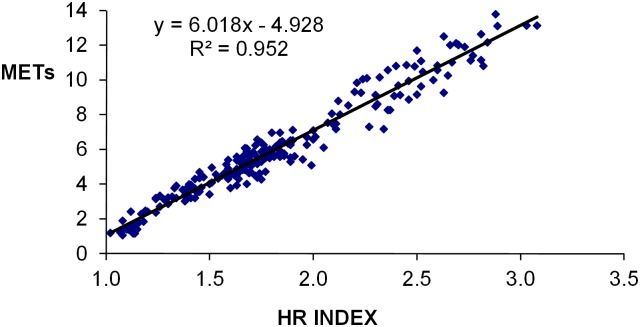
Linear regression plot of HR index equation. An analysis from data (n = 220) derived from 60 studies with the HR index equation simplified to METs = 6 x HR index– 5.

Cardiovascular pathology frequently screened for with treadmill testing includes both CAD and CHF. In using CRF as an outcome measure from a treadmill test, VO_2peak_ is commonly expressed as METs with 1 MET being the VO_2_ at rest with current convention stating that it is equal to 3.5 mL O_2_ ·kg^-1^·min^-1^ [29]. Kaplan-Meier curves have been used extensively to document the link between CRF and long-term morbidity/mortality [30,31]. Although VO_2_ can be predicted from treadmill speed, incline or the test time for a particular protocol, currently the only way to ensure an accurate measurement of VO_2_ is direct measurement with gas analysis. Using only two simple measurements, rest HR and an activity HR (either sub-maximal or maximal), the recently published HR index (HRI = activity HR/rest HR), equation for predicting VO_2_ expressed as METs is associated with a high correlation between HRI and VO_2_, the equation being METs = 6 x HRI -5 [32]. The HRI equation was derived from group mean data from 60 studies in which an exercise test contained a resting HR (HR_rest_), and a VO_2_ measured at the activity HR (either submaximal or peak) and expressed in the form of mLO_2_ ·kg^-1^·min^-1^ or METs. The original data are shown as a regression plot in Fig 1. The utility of this equation is that it provides a simple independent surrogate method of estimating VO_2_ using only the rest and either the sub-maximal or maximal activity HR measurements. Though the HRI equation was developed from aggregate data, there has been no analysis to date that has established its predictive accuracy for assessment of VO_2_.

The objective of this study was to compare aggregate HRI-derived VO_2_ (HRI-VO_2_) data against VO_2peak_ from two different treadmill tests, either: 1) VO_2_ measured with conventional gas analysis equipment (TM-VO_2meas_) or 2) VO_2_ predicted from equations based on treadmill speed, incline or treadmill time (TM-VO_2pred_).

## Methods

### Study selection

Treadmill studies involving assessment of VO_2peak_, reporting either TM-VO_2meas_ or TM-VO_2pred_, were identified through a systematic search conducted on at least a monthly basis from October 2011 till March 2013 using MEDLINE, Google Scholar and Web of Science. Search terms included (in various combinations) exercise testing, oxygen uptake, VO_2_, CRF, cardiovascular disease (CVD), CAD, CHF and physical activity. With publications having the prerequisite HR data extensive cross-referencing was undertaken to source other publications with eligible criteria [33].

Eligibility criteria for study inclusion are 1) >100 patients enrolled, 2) documented VO_2peak_ (either measured or predicted) expressed as either mL O_2_ ·kg^-1^·min^-1^ or as METs, 3) measured maximal HR (HR_max_) associated with VO_2peak_, and 4) measured HR_rest_. Where large scale studies included cycle ergometry in conjunction with treadmill testing, the study was excluded. In publications likely to have used a similar subject cohort based on 1. participating authors, 2. study location, 3. time period when the study was performed and 4. characteristics of the study population e.g. healthy, suspected or known CAD the most recent publication was chosen. From the HR data, a predicted MET value (VO_2peak_) was derived using the HRI equation (METs = 6 x HR index– 5, where HR index is HR_max_/HR_rest_).

At the time of closure of data acquisition in March 2013 a total of 40 studies (TM-VO_2meas_; n = 20 studies, TM-VO_2pred_; n = 20 studies) had been identified with all but one being published since 1991. MEDLINE searching identified 19 of the 40 studies (TM-VO_2meas_; n = 11 studies, TM-VO_2pred_; n = 8 studies) used in this analysis with the remaining 21 studies being sourced through Web of Science, Google Scholar and cross referencing. The TM-VO_2meas_ studies had a bias towards clinical outcomes related to CHF whereas the TM-VO_2pred_ studies were frequently associated with long-term outcome (survival) in screening for CVD. Though multiple search strategies were used to obtain studies meeting selection criteria it is acknowledged that even with rigorous attention to search detail, suitable studies may have been missed.

**Fig 2:**
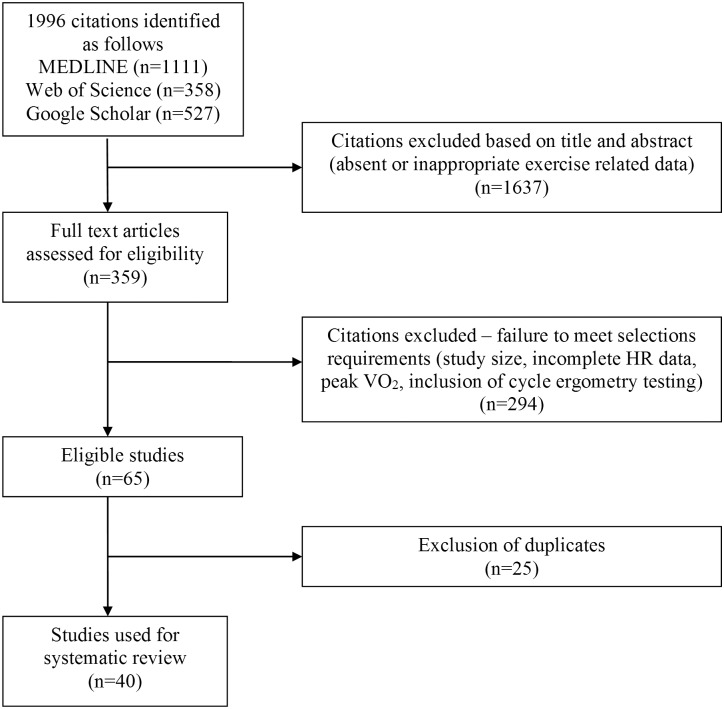
Study selection process used for data acquisition.

Fig 2 details the study selection process at the completion of data acquisition in March 2013.

### Statistical analysis

Categorical variables were expressed as numbers and percentages with continuous variables expressed as mean ± standard deviation. Student’s paired t-test was used to compare HRI-VO_2_ against both TM-VO_2meas_ and TM-VO_2pred_. Results are expressed in two formats, namely 1) pooled data for each of TM-VO_2meas_ and TM-VO_2pred_ against HRI-VO_2_ expressed as group means and shown in the form of line of identity and Bland Altman plots [34] and 2) CRF data shown in tertiles for both TM-VO_2meas_ and TM-VO_2pred_ groups against HRI-VO_2_.

## Results

### Studies used in the analyses

There were 11,477 subjects in the 20 TM-VO_2meas_ studies (range 110 to 4631, median 337) and, with each study mean VO_2meas_ value representing a data point, there was a total of 45 data points. There was a considerably larger number of subjects at 105,044 (range 772 to 22,275, median 3,736) in the 20 TM-VO_2pred_ studies and, with each study mean VO_2pred_ value representing a data point, there were 57 data points. Age and gender distribution was similar for the TM-VO_2meas_ (51.0 years and 64.9% males) and TM-VO_2pred_ groups (52.9 years and 71.0% males).

The principal details of the 40 treadmill studies used in the analysis are outlined in Table 1. These include the test protocol, use of handrail support and the health status of participants. Of the 20 TM-VO_2meas_ studies, 14 (70%) involved subjects with CHF and all 14 used protocols other than the standard Bruce protocol [35]. The design of these alternate protocols reduced the stage increment of VO_2_ usually to 2 METs or less with certain ramp protocols having increments of less than 1 MET per minute. In only two of the TM-VO_2meas_ studies was hand rail support mentioned, being ‘not permitted’ in one study (Dressendorfer [36]) and ‘discouraged’ in the other (Oliveira [37]).

**Table 1:** Description of studies, patient diagnosis, and test protocol in which oxygen uptake was either measured or predicted using a prediction equation (Pred EQ).

First Author	Year	n	Age (years)	Male%	Category	Test	Rail support	Pred EQ
***Measured VO_2_***								
Bard	2006	355	51	72	CHF	ramp	ns	
Diller	2006	727	33	52	ACHD	MB	ns	
Dressendorfer	1993	182	57	100	CAD	MB	NP	
Elmariah	2006	594	52	72	CHF	ramp	ns	
Harrington	1997	131	59	100	CHF, H	MB	ns	
Ingle	2007	394	65	74	CHF	MB	ns	
Jorde	2008	278	52	77	CHF	Na	ns	
Kohrt	1991	110	64	50	H	B,O	ns	
Kubrychtova	2009	712	56	72	CHF	O	ns	
Lanier	2012	320	52	75	CHF	Na	ns	
McDonough	1970	144	51	100	H	B	ns	
Nes	2012	4631	48	49	H	ramp	ns	
Oliveira	2009	948	57	100	CPD, H	ramp	DIS	
Osada	1998	154	52	75	CHF	MB, MNa	ns	
Peterson	2003	369	51	72	CHF	O	ns	
Robbins	1999	487	52	71	CHF, H	Na	ns	
Schalcher	2003	146	52	88	CHF	ramp	ns	
Stolker	2006	221	49	68	CHF	O	ns	
Williams	2001	219	56	76	CHF	B, MB	ns	
Witte	2006	355	66	68	CHF, H	MB	ns	
***Predicted VO_2_***								
Adabag	2008	12555	46	100	CAD**°**	B	ns	EQ-S
Aijaz	2008	10897	54	75	CVD, CVD**°**	B	ns	ns
Arruda-Olson	2002	5798	62	57	CAD, CAD^**?**^	B, MB, Na	ns	ns
Carnethon	2003	4487	25	45	H	MBa	ns	ns
Cheng	2003	2333	49	100	DM	MBa	ns	EQ-S
Elhendy	2001	1618	55	35	CAD^**?**^	B, MB, Na	ns	ns
Gulati	2010	5437	52	0	CAD**°**	B	LS	EQ-R
Kim	2007	22275	51	59	CVD**°**	B, MB, O	NP	EQ-R
Kokkinos	2009	4631	61	100	HT	B, ramp	DIS	EQ-R
Lai	2004	5625	59	100	CVD**°**	ramp, O	ns	EQ-R
Lauer	1999	2953	58	64	CVD**°**, CVD^**?**^	B, MB	NP	EQ-R
Lipinski	2005	1914	52	100	CAD, CHF, CAD**°**	ramp, O	ns	ns
Mahenthiran	2005	1268	60	52	CAD, CAD**°**	B	ns	ns
McAuley	2007	6876	58	97	CAD, CAD**°**	ramp	DIS	EQ-R
Mora	2003	2985	47	0	CAD**°**	B	ns	EQ-R
Morrow	1993	2546	59	100	CAD°, CAD, CHF	ramp, O	ns	EQ-R
Myers	2002	6213	59	100	CAD, CAD**°**	ramp	DIS	EQ-R
Negishi	2013	914	56	56	DM	B, MB	NP	EQ-R
Peteiro	2010	2947	62	61	CAD, CAD^**?**^	B, MB, Na	ns	ns
Shaw	2011	772	63	0	CAD^**?**^	B, MB	ns	ns

References are available in the supplementary digital content. **Category:** ACHD, adult congenital heart disease; CAD, coronary artery disease (CAD**°**, absent; CAD^**?**^, suspected); CHF, congestive heart failure; CPD, cardiopulmonary disease; CVD, cardiovascular disease; DM, diabetes mellitus; H, healthy; HT, hypertension. **Treadmill test:** B, Bruce protocol; Ba, Balke protocol; Na, Naughton protocol; ramp, ramp protocol; M, modified protocol; O, other protocol; **Rail support**: ns, not stated; NP, not permitted; DIS, discouraged; LS, light support; **Equation**: EQ-S, stated equation; EQ-R, referenced equation; ns, not stated.

Typically, subjects with known or suspected CVD or with significant cardiovascular risk factors were involved in the TM-VO_2pred_ studies (Table 1). A Bruce protocol, either as the standard or a modified protocol, was used in 13 (65%) of the 20 TM-VO_2pred_ studies. With TM-VO_2pred_ studies, the use of handrail support was defined in seven studies (35%) and not stated in the remaining 13 studies. Descriptors of handrail support used for these seven studies were ‘discouraged’ in 3 studies, ‘not permitted’ in 3 studies and ‘light hand rail support’ in 1 study. Predictive treadmill equations in TM-VO_2pred_ studies were either given or referenced in only 12 (60%) of the 20 studies.

### Characterization of study groups

#### A. Group means: oxygen consumption and heart rate

The mean TM-VO_2pred_ reported in the 20 studies was 8.12 METS; the mean TM-VO_2meas_ reported in the 20 studies was 6.51 METS, a difference of 1.61 Mets or 24.7% (Table 2). The mean HR_rest_ with TM-VO_2pred_ was 75.6 beats∙min^-1^ and with TM-VO_2meas_ was 77.6 beats∙min^-1^; the mean HR_max_ for TM-VO_2pred_ 146.3 beats∙min^-1^ and TM-VO_2meas_ 147.1 beats∙min^-1^ (Table 2). However, the absolute differences in group means for HR_rest_ and HR_max_ between TM-VO_2pred_ and TM-VO_2meas_ were small at 2.0 beats∙min^-1^ for HR_rest_ and only 0.8 beat∙min^-1^ for HR_max_ (Table 2).

**Table 2:** Heart rate and oxygen consumption data for TM-VO_2meas_ and TM-VO_2pred_. Group mean (± 1SD) heart rate (HR) and oxygen consumption (VO_2_) data. HR_rest_, HR_peak_, HRI-VO_2_ and VO_2peak_ for TM-VO_2meas_ and TM-VO_2pred_.

	Studies	Data points	HR_rest_ beats∙min^-1^	HR_peak_ beats∙min^-1^	VO_2peak_ METs	HRI-VO_2_ METs
**TM-VO**_**2pred**_	20	57	75.6 ± 5.3	146.3 ± 16.6	8.12 ± 1.85	6.71 ± 1.92
**TM-VO**_**2meas**_	20	45	77.6 ± 7.7	147.1 ± 18.8	6.51 ± 2.25	6.54 ± 2.28

Alternatively if VO_2peak_ is determined by HRI-VO_2_ the difference between TM-VO_2pred_ and TM-VO_2meas_ is reduced to only 0.17 MET or 2.6% (TM-VO_2pred_ 6.71 METs, TM-VO_2meas_ 6.54 METs), a not unexpected result in view of the small differences in HR_rest_ and HR_max_ between these two groups (Table 2).

#### B. Comparison of measured VO_2_ and predicted VO_2_ versus VO_2_ predicted by HRI

**Fig 3:**
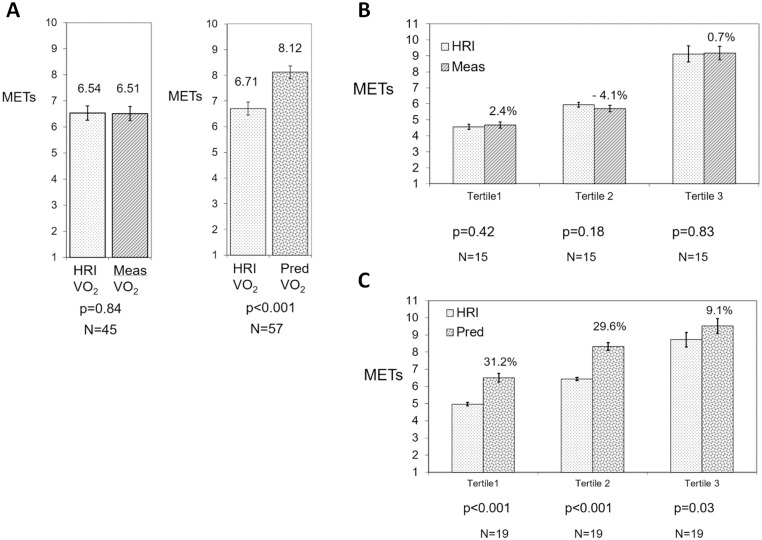
Comparison of pooled data from 20 studies for TM-VO_2meas_ and TM-VO_2pred_ against HRI-VO_2_. A. Comparison of group mean data for 20 TM-VO_2meas_ and TM-VO_2pred_ studies against HRI-VO2 (mean ± SE), B. Comparison of cardiorespiratory fitness tertiles from 20 studies for TM-VO_2meas_ against HRI-VO_2_ (mean ±SE). Percentage difference between TM-VO_2meas_ and HRI-VO_2_ shown within figure and C. Comparison of cardiorespiratory fitness tertiles from 20 studies for TM-VO_2pred_ against HRI-VO_2_ (mean ±SE). Percentage difference between TM-VO_2pred_ and HRI-VO_2_ shown within figure.

When using the HRI to calculate VO_2peak_, there was no significant difference (0.4%, p = 0.84) in the pooled VO_2_ data with mean (± SD) MET values of 6.51(±2.25) for TM-VO_2meas_ and 6.54 (±2.28) for HRI-VO_2_ (Fig 3A). However, a highly significant difference (21.1%, p<0.001) was seen between TM-VO_2pred_ and HRI-VO_2_ with respective values of 8.12 (±1.85) METs and 6.71 (±1.92) METs (Fig 3A).

Even when expressed in tertiles based on HRI-VO_2_, there were no significant differences between TM-VO_2meas_ and HRI-VO_2_ by VO_2_ tertile; tertile 1, 2.4% (p = 0.42), tertile 2, -4.1% (p = 0.18) and tertile 3, 0.7% (p = 0.83) (Fig 3B). By comparison, each tertile for the TM-VO_2pred_ groups showed a significant difference from HRI-VO_2_; tertile 1, 31.2% (p<0.001), tertile 2, 29.6% (p<0.001) and tertile 3, 9.1% (p = 0.03) (Fig 3C).

**Fig 4:**
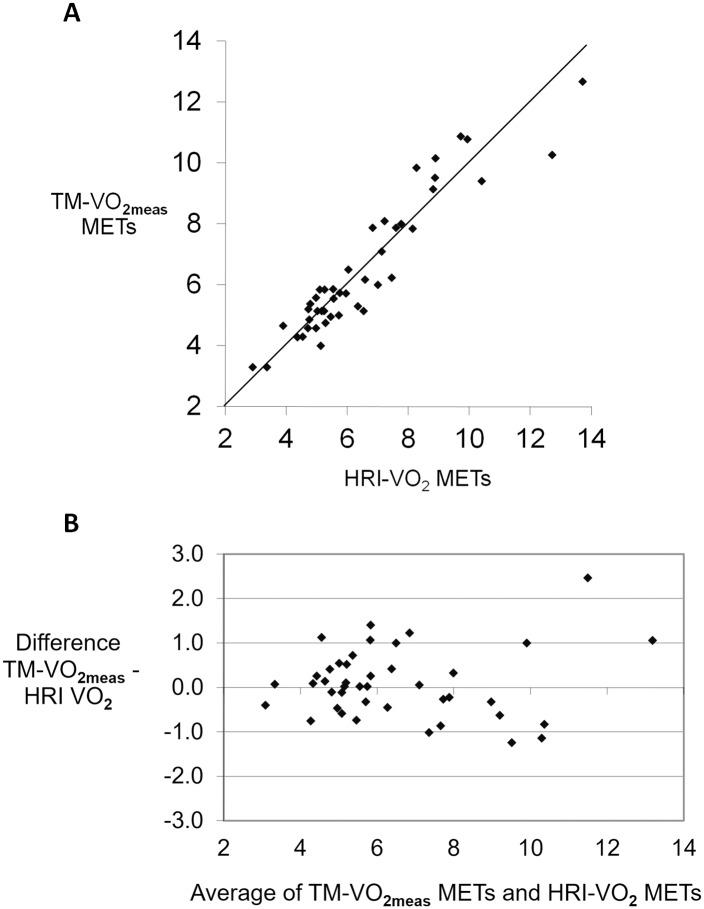
Line of identity and Bland Altman plot for TM-VO_2meas_. A. Line of identity for TM-VO_2meas_ and B. against Bland Altman plot for TM-VO_2meas_ against HRI-VO_2_.

**Fig 5:**
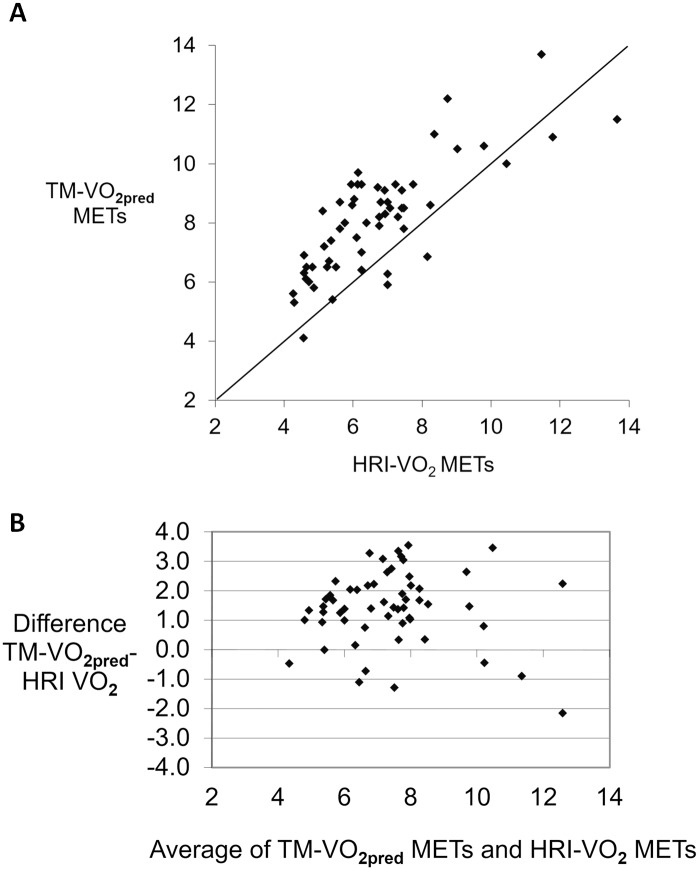
Line of identity and Bland Altman plot for TM-VO_2pred_. **A.** Line of identity for TM-VO_2pred_ and B. against Bland Altman plot for TM-VO_2pred_ against HRI-VO_2_.

The plot of TM-VO_2meas_ against HRI-VO_2_ shows a uniform distribution around the line of identity with the Bland Altman plot suggesting that there is no bias between these two separate methods of determining VO_2peak_ (Fig 4A and 4B). However, a similar line of identity plot for TM-VO_2pred_ against HRI-VO_2_ indicates a strong bias with the Bland Altman plot indicating a systematic error in support of over-prediction of TM-VO_2pred_ (Fig 5A and 5B).

## Discussion

It is crucial to have high quality CRF data for use in epidemiological studies as management strategies involving both pharmacological and lifestyle intervention rely on this accuracy. The utility of the HRI equation [32] as a surrogate measure of VO_2_ expressed in METs is confirmed in this study when assessed against VO_2peak_ for both TM-VO_2meas_ measured with conventional gas analysis equipment and for TM-VO_2pred_ predicted from equations based on treadmill speed, incline or treadmill time. A close agreement between HRI-VO_2_ and TM-VO_2meas_ was observed in the 20 TM-VO_2meas_ studies with only a 0.4% difference (p = 0.84) between group means. By comparison, a highly significant 21.1% (p<0.001) over-prediction of VO_2peak_ was observed when comparing HRI-VO_2_ against TM-VO_2pred_ in the 20 TM-VO_2pred_ studies. The magnitude of the potential error using TM-VO_2pred_ challenges the current methods of treadmill prediction of CRF which appear to lead to overestimation of CRF and potentially to false prognostic classification.

If the magnitude of the disparity between HRI-VO_2_ and TM-VO_2pred_ as shown in this study is, for example, applied to the outcome data of CRF as expressed in METs in the meta-analysis by Kodama [2], there is a strong likelihood of a false classification based on the over-prediction of CRF. For example, in treadmill studies investigating the effect of handrail support, a practice that lengthens treadmill time, VO_2peak_ is over-predicted by 20% to 30% [9–13,17] which would lead to a potentially false prognostic classification of CRF. To correct for the consistently observed over-prediction of VO_2peak_ of around 20% resulting from the use of handrail support, Foster has developed simple modifications of the ACSM equations for use when handrail support is observed during treadmill testing [17]. None of the 20 TM-VO_2pred_ studies used in this analysis referenced use of the Foster or similar equations to correct for observed handrail support. This prediction error could potentially apply to other published studies that express results in the form of survival tables and Kaplan-Meier curves. The measurement of CRF is not only limited to CVD. CRF also defines long-term risk in both healthy subjects and other common medical conditions, such as stroke [38], dementia [39] and diabetes mellitus [40]. In the TM-VO_2pred_ group of studies, the smallest difference (9.1%) between HRI-VO_2_ and TM-VO_2pred_ was observed in the highest CRF tertile. Presumably, the fittest subjects find less difficulty with treadmill walking and so have less need for handrail support. Conversely, the least fit, i.e., the lowest tertile, are most likely to utilize handrail support, even when instructed otherwise, and, in the present study, they demonstrated a 31.2% difference between HRI-VO_2_ and TM-VO_2pred_. Results from the HUNT 3 Fitness Study also noted the greatest overestimation of VO_2peak_ in the least fit subjects [18].

Collectively the 20 TM-VO_2pred_ studies used in this analysis involve a tenfold greater number of subjects when compared with the 20 TM-VO_2meas_ studies, whether considering the total number of subjects (105,044 TM-VO_2pred_ versus 11,477 TM-VO_2meas_) or the median number (3,736 TM-VO_2pred_ versus 337 TM-VO_2meas_). This observation indicates an inherent bias in using predicted VO_2_ studies for epidemiological purposes. In recognizing the need for high quality population CRF data, the Fitness Registry and the Importance of Exercise: A National Database (FRIEND) was established in 2014 [41]. A recent publication from this group has provided age-related reference standards of CRF from 7783 tests in which VO_2max_ was determined by gas analysis, the authors highlighting the shortcomings of using TM-VO_2pred_ largely because of over-prediction of VO_2max_ associated with hand rail support [42]. Their statement together with the observations in the present review suggest that, for the continued use of TM-VO_2pred_ data, a reappraisal of current methods used for prediction of VO_2peak_ warrants consideration.

One important question arising from this analysis is the value of using maximal HRI to predict VO_2peak_ from HR derived values (rest and peak) as opposed to treadmill parameters (speed, incline or treadmill time). When calculating maximal HRI, two independent predictors of future CVD risk, namely an estimated VO_2peak_ [2,43] and HR_rest_ [44] are incorporated within the HRI. The maximal HRI is based on two measured values of HR and, when used as an index, there is minimal predictive error especially when compared to VO_2pred_ using equations based on speed, incline or treadmill time. As a 1.0 MET increment corresponds to a HRI increment of 0.167, Kaplan-Meier curves ranging from <5 to >10 METs have a corresponding HRI range from <1.67 to >2.50 (e.g., 5 METs = Rest [HRI = 1] + 4 METs [HRI = 4 x 0.167] = 1.67). In considering a range of activity from rest (1.0 MET) to the maximum aerobic performance of an elite athlete (e.g. 19 METs), the corresponding range of HRI would be from 1 to 4. The simplicity of calculating HRI together with the range of index used for clinical evaluation suggests that it could provide a useful addition to the assessment of CRF. To illustrate this, a range of 5, 10 and 15 MET levels have corresponding HRIs of 1.67, 2.5 and 3.33.

## Study Limitations

This review has used the simple concept of HRI as a surrogate measure of VO_2_. The equation was established from aggregate data acquired from 60 studies. In applying the HR index to this analysis, we have compared aggregate data from TM-VO_2pred_ and TM-VO_2meas_ against HRI-VO_2_ with no intention of indicating the individual predictive accuracy of the equation. Ideally the use of individual, as opposed to aggregate data would have been preferable but it was beyond the capability of this analysis.

## Conclusions

The usefulness of CRF is well established for assessing CV risk with treadmill testing providing a simple and convenient method of assessing CRF. The aggregate analysis used in this study shows a close relationship, i.e., a non-significant 0.4% difference, between HRI-VO_2_ and TM-VO_2meas_ but a large and highly significant 21.1% difference between HRI-VO_2_ and TM-VO_2pred_.This overestimation of TM-VO_2pred_, and so CRF, challenges the validity of predicting VO_2 peak_ from equations based on treadmill speed, incline or protocol time when attempting to document a link between CRF and long-term morbidity/mortality.

## Supporting Information

S1 File Supplementary Reference List– 40 treadmill studies. File listing the 40 treadmill studies used for analysis.
(RTF)
