# Supplementary material for: How Accurate Is the Prediction of Maximal Oxygen Uptake with Treadmill Testing?
Source: PLoS One. 2016 Nov 22;11(11):e0166608. doi: 10.1371/journal.pone.0166608 (PMC5119771; doi:10.1371/journal.pone.0166608)
Supplement: S1 File — File listing the 40 treadmill studies used for analysis. (RTF) [file pone.0166608.s001.rtf]

Supplementary reference list – 40 treadmill studies

1. 	Adabag AS, Grandits GA, Prineas RJ, Crow RS, Bloomfield HE, Neaton JD, et al. (2008) Relation of heart rate parameters during exercise test to sudden death and all-cause mortality in asymptomatic men. Am J Cardiol 101: 1437-1443.
2. 	Aijaz B, Babuin L, Squires RW, Kopecky SL, Johnson BD, Thomas RJ, et al. (2008) Long-term mortality with multiple treadmill exercise test abnormalities: comparison between patients with and without cardiovascular disease. Am Heart J 156: 783-789.
3.	 Arruda-Olson AM, Juracan EM, Mahoney DW, McCully RB, Roger VL, Pellikka PA (2002) Prognostic value of exercise echocardiography in 5,798 patients: is there a gender difference? J Am Coll Cardiol 39: 625-631.
4. 	Bard RL, Gillespie BW, Clarke NS, Egan TG, Nicklas JM (2006) Determining the best ventilatory efficiency measure to predict mortality in patients with heart failure. J Heart Lung Transplant 25: 589-595.
5. 	Carnethon MR, Gidding SS, Nehgme R, Sidney S, Jacobs Jr DR, Liu K (2003) Cardiorespiratory fitness in young adulthood and the development of cardiovascular disease risk factors. JAMA 290: 3092-3100.
6. 	Cheng YJ, Lauer MS, Earnest CP, Church TS, Kampert JB, Gibbons LW, et al. (2003) Heart rate recovery following maximal exercise testing as a predictor of cardiovascular disease and all-cause mortality in men with diabetes. Diabetes Care 26: 2052-2057.
7. 	Diller G-P, Dimopoulos K, Okonko D, Uebing A, Broberg CS, Babu-Narayan S, et al. (2006) Heart rate response during exercise predicts survival in adults with congenital heart disease. J Am Coll Cardiol 48: 1250-1256.
8. 	Dressendorfer RH, Franklin BA, Gordon S, Timmis GC (1993) Resting oxygen uptake in coronary artery disease. Influence of chronic beta-blockade. Chest 104: 1269-1272.
9. 	Elhendy A, Shub C, McCully RB, Mahoney DW, Burger KN, Pellikka PA (2001) Exercise echocardiography for the prognostic stratification of patients with low pretest probability of coronary artery disease. Am J Med 111: 18-23.
10. 	Elmariah S, Goldberg LR, Allen MT, Kao A (2006) Effects of gender on peak oxygen consumption and the timing of cardiac transplantation. J Am Coll Cardiol 47: 2237-2242.
11. 	Gulati M, Shaw LJ, Thisted RA, Black HR, Bairey Merz CN, Arnsdorf MF (2010) Heart rate response to exercise stress testing in asymptomatic women: the St. James women take heart project. Circulation 122: 130-137.
12. 	Harrington D, Anker SD, Chua TP, Webb-Peploe KM, Ponikowski PP, Poole-Wilson PA, et al. (1997) Skeletal muscle function and its relation to exercise tolerance in chronic heart failure. J Am Coll Cardiol 30: 1758-1764.
13. 	Ingle L, Goode K, Carroll S, Sloan R, Boyes C, Cleland JGF, et al. (2007) Prognostic value of the VE/VCO2 slope calculated from different time intervals in patients with suspected heart failure. Int J Cardiol 118: 350-355.
14. 	Jorde UP, Vittorio TJ, Kasper ME, Arezzi E, Colombo PC, Goldsmith RL, et al. (2008) Chronotropic incompetence, beta-blockers, and functional capacity in advanced congestive heart failure: time to pace? Eur J Heart Fail 10: 96-101.
15. 	Kim ESH, Ishwaran H, Blackstone E, Lauer MS (2007) External prognostic validations and comparisons of age- and gender-adjusted exercise capacity predictions. J Am Coll Cardiol 50: 1867-1875.
16. 	Kohrt WM, Malley MT, Coggan AR, Spina RJ, Ogawa T, Ehsani A, et al. (1991) Effects of gender, age, and fitness level on response of VO2max to training in 60-71 yr olds. J Appl Physiol 71: 2004-2011.
17. 	Kokkinos P, Manolis A, Pittaras A, Doumas M, Giannelou A, Panagiotakos DB, et al. (2009) Exercise capacity and mortality in hypertensive men with and without additional risk factors. Hypertension 53: 494-499.
18. 	Kubrychtova V, Olson TP, Bailey KR, Thapa P, Allison TG, Johnson BD (2009) Heart rate recovery and prognosis in heart failure patients. Eur J Appl Physiol 105: 37-45.
19. 	Lai S, Kaykha A, Yamazaki T, Goldstein M, Spin JM, Myers J, et al. (2004) Treadmill scores in elderly men. J Am Coll Cardiol 43: 606-615.
20. 	Lanier GM, Zheng Q, Wagman G, Tseng CH, Myers JN, Vittorio TJ (2012) Simple prediction formula for peak oxygen consumption in patients with chronic heart failure. J Exerc Sci Fit: 23-27.
21. 	Lauer MS, Francis GS, Okin PM, Pashkow FJ, Snader CE, Marwick TH (1999) Impaired chronotropic response to exercise stress testing as a predictor of mortality. JAMA 281: 524-529.
22. 	Lipinski MJ, Vetrovec GW, Gorelik D, Froelicher VF (2005) The importance of heart rate recovery in patients with heart failure or left ventricular systolic dysfunction. J Card Fail 11: 624-630.
23. 	Mahenthiran J, Bangalore S, Yao SS, Chaudhry FA (2005) Comparison of prognostic value of stress echocardiography versus stress electrocardiography in patients with suspected coronary artery disease. Am J Cardiol 96: 628-634.
24. 	McAuley P, Myers J, Abella J, Froelicher V (2007) Body mass, fitness and survival in veteran patients: another obesity paradox? Am J Med 120: 518-524.
25. 	McDonough JR, Kusumi F, Bruce RA (1970) Variations in maximal oxygen intake with physical activity in middle-aged men. Circulation 41: 743-751.
26. 	Mora S, Redberg RF, Cui Y, Whiteman MK, Flaws JA, Sharrett AR, et al. (2003) Ability of exercise testing to predict cardiovascular and all-cause death in asymptomatic women. JAMA 290: 1600-1607.
27. 	Morrow K, Morris CK, Froelicher VF, Hideg A, Hunter D, Johnson E, et al. (1993) Prediction of cardiovascular death in men undergoing noninvasive evaluation for coronary artery disease. Ann Intern Med 118: 689-695.
28. 	Myers J, Prakash M, Froelicher V, Do D, Partington S, Atwood JE (2002) Exercise capacity and mortality among men referred for exercise testing. N Engl J Med 346: 793-801.
29. 	Negishi K, Seicean S, Negishi T, Yingchoncharoen T, Aljaroudi W, Marwick TH (2013) Relation of heart-rate recovery to new onset heart failure and atrial fibrillation in patients with diabetes mellitus and preserved ejection fraction. Am j Cardiol 111: 748-753.
30. 	Nes BM, Janszky I, Aspenes ST, Bertheussen GF, Vatten LJ, Wisløff U (2012) Exercise patterns and peak oxygen uptake in a healthy population: the HUNT study. Med Sci Sports Exerc 44: 1881-1889.
31. 	Oliveira RB, Myers J, Araujo CGS, Abella J, Mandic S, Froelicher V (2009) Maximal exercise oxygen pulse as a predictor of mortality among male veterans referred for exercise testing. Eur J Cardiovasc Prev Rehabil 16: 358-364.
32. 	Osada N, Chaitman BR, Miller LW, Yip D, Cishek MB, Wolford TL, et al. (1998) Cardiopulmonary exercise testing identifies low risk patients with heart failure and severely impaired exercise capacity considered for heart transplantation. J Am Coll Cardiol 31: 577-582.
33. 	Peteiro J, Bouzas-Mosquera A, Broullón FJ, Garcia-Campos A, Pazos P, Castro-Beiras A (2010) Prognostic value of peak and post-exercise treadmill exercise echocardiography in patients with known or suspected coronary artery disease. Eur Heart J 31: 187-195.
34. 	Peterson LR, Schechtman KB, Ewald GA, Geltman EM, Meyer T, Krekeler P, et al. (2003) The effect of beta-adrenergic blockers on the prognostic value of peak exercise oxygen uptake in patients with heart failure. J Heart Lung Transplant 22: 70-77.
35. 	Robbins M, Francis G, Pashkow FJ, Snader CE, Hoercher K, Young JB, et al. (1999) Ventilatory and heart rate responses to exercise: better predictors of heart failure mortality than peak oxygen consumption. Circulation 100: 2411-2417.
36. 	Schalcher C, Rickli H, Brehm M, Weilenmann D, Oechslin E, Kiowski W, et al. (2003) Prolonged oxygen uptake kinetics during low-intensity exercise are related to poor prognosis in patients with mild-to-moderate congestive heart failure. Chest 124: 580-586.
37. 	Shaw LJ, Mieres JH, Hendel RH, Boden WE, Gulati M, Veledar E, et al. (2011) Comparative effectiveness of exercise electrocardiography with or without myocardial perfusion single photon emission computed tomography in women with suspected coronary artery disease clinical perspective results from the What is the Optimal Method for ischemia Evaluation in Women (WOMEN) Trial. Circulation 124: 1239-1249.
38. 	Stolker JM, Heere B, Geltman EM, Schechtman KB, Peterson LR (2006) Prospective comparison of ventilatory equivalent versus peak oxygen consumption in predicting outcomes of patients with heart failure. Am J Cardiol 97: 1607-1610.
39. Williams S, Cooke G, Wright D, Parsons W, Riley R, Marshall P, et al. (2001) Peak exercise cardiac power output; a direct indicator of cardiac function strongly predictive of prognosis in chronic heart failure. Eur Heart J 22: 1496-1503.
40. Witte KKA, Cleland JGF, Clark AL (2006) Chronic heart failure, chronotropic incompetence, and the effects of beta blockade. Heart 92: 481-486.
